# Shaping the tripartite symbiosis: are termite microbiome functions directed by the environmentally acquired fungal cultivar?

**DOI:** 10.1186/s42523-024-00332-5

**Published:** 2024-08-06

**Authors:** Robert M. Murphy, Veronica M. Sinotte, Ana Cuesta-Maté, Justinn Renelies-Hamilton, Mikael Lenz-Strube, Michael Poulsen

**Affiliations:** 1 Section for Ecology and Evolution, Department of Biology, University of Copenhagen, Copenhagen East, Denmark; 2 Center for Evolutionary Hologenomics, Globe Institute, University of Copenhagen, Copenhagen K, Denmark; 3 Jane Goodall Institute Spain and Senegal, Dindefelo Biological Station, Dindefelo, Kedougou Senegal; 4 Center for Microbial Secondary Metabolites, Technical University of Denmark, Kongens Lyngby, Denmark

**Keywords:** Symbiosis, Macrotermitinae, *Termitomyces*, Gut microbiome, Metagenomes, Horizontal transmission, Amplicon sequencing, AZCL, CAZyme

## Abstract

Microbiome assembly critically impacts the ability of hosts to access beneficial symbiont functions. Fungus-farming termites have co-evolved with a fungal cultivar as a primary food source and complex gut microbiomes, which collectively perform complementary degradation of plant biomass. A large subset of the bacterial community residing within termite guts are inherited (vertically transmitted) from parental colonies, while the fungal symbiont is, in most termite species, acquired from the environment (horizontally transmitted). It has remained unknown how the gut microbiota sustains incipient colonies prior to the acquisition of the fungal cultivar, and how, if at all, bacterial contributions are modulated by fungus garden establishment. Here, we test the latter by determining the composition and predicted functions of the gut microbiome using metabarcoding and shotgun metagenomics, respectively. We focus our functional predictions on bacterial carbohydrate-active enzyme and nitrogen cycling genes and verify compositional patterns of the former through enzyme activity assays. Our findings reveal that the vast majority of microbial functions are encoded in the inherited microbiome, and that the establishment of fungal gardens incurs only minor modulations of predicted bacterial capacities for carbohydrate and nitrogen metabolism. While we cannot rule out that other symbiont functions are gained post-fungus garden establishment, our findings suggest that fungus-farming termite hosts are equipped with a near-complete set of gut microbiome functions at the earliest stages of colony life. This inherited, incipient bacterial microbiome likely contributes to the high extent of functional specificity and coevolution observed between termite hosts, gut microbiomes, and the fungal cultivar.

**Supplementary Information:**

The online version contains supplementary material available at 10.1186/s42523-024-00332-5.

## Introduction

Bacteria and fungi frequently form mutualistic symbioses with eukaryotes for host nutrition, where symbionts enable the utilization of otherwise inaccessible nutrient sources or supplement nutritionally deficient diets [1, 2]. The assembly and maintenance of complex microbiomes are thus critical for community functions and services to hosts. As has been observed in a multitude of studies, a balanced microbiome is undeniably important for health [3–7], immune system maturation [5, 6, 8, 9], and normal development [5, 6, 9, 10] in a broad range of hosts. Rather than being static, a beneficial microbiome supports its host differently across developmental stages [6, 10], as evident from age-directed compositional variance observed in humans [11] and pigs [12]. Compositional changes early in life are typically prompted by major shifts in diet, such as weaning in mammals [9, 10, 13, 14]. Such shifts also alter the functional capabilities of microbiomes. In humans, infant microbiomes are dominated by milk oligosaccharide processing bacteria such as *Bifidobacterium*, *Bacteroides*, and others prior to weaning, followed by a maturation into to an adult microbiome dominated by Firmicutes and Bacteroidetes with digestive and other roles [7, 9]. Our understanding of how host development directs changes in microbiomes predominantly comes from work on mammals, but other animals also experience shifts in diet at critical stages in life. For example, the tripartite fungus-farming termite (Macrotermitinae) symbiosis involves complex bacterial microbiomes [15, 16] and a basidiomycete fungus cultivated as the primary food source for the termite host [17]. In most termite species, this fungal cultivar is absent for the first several months of colony life, after which termite hosts transition to a fungal diet. Yet, the consequences of this shift for bacterial symbiont roles are not known.

**Fig. 1:**
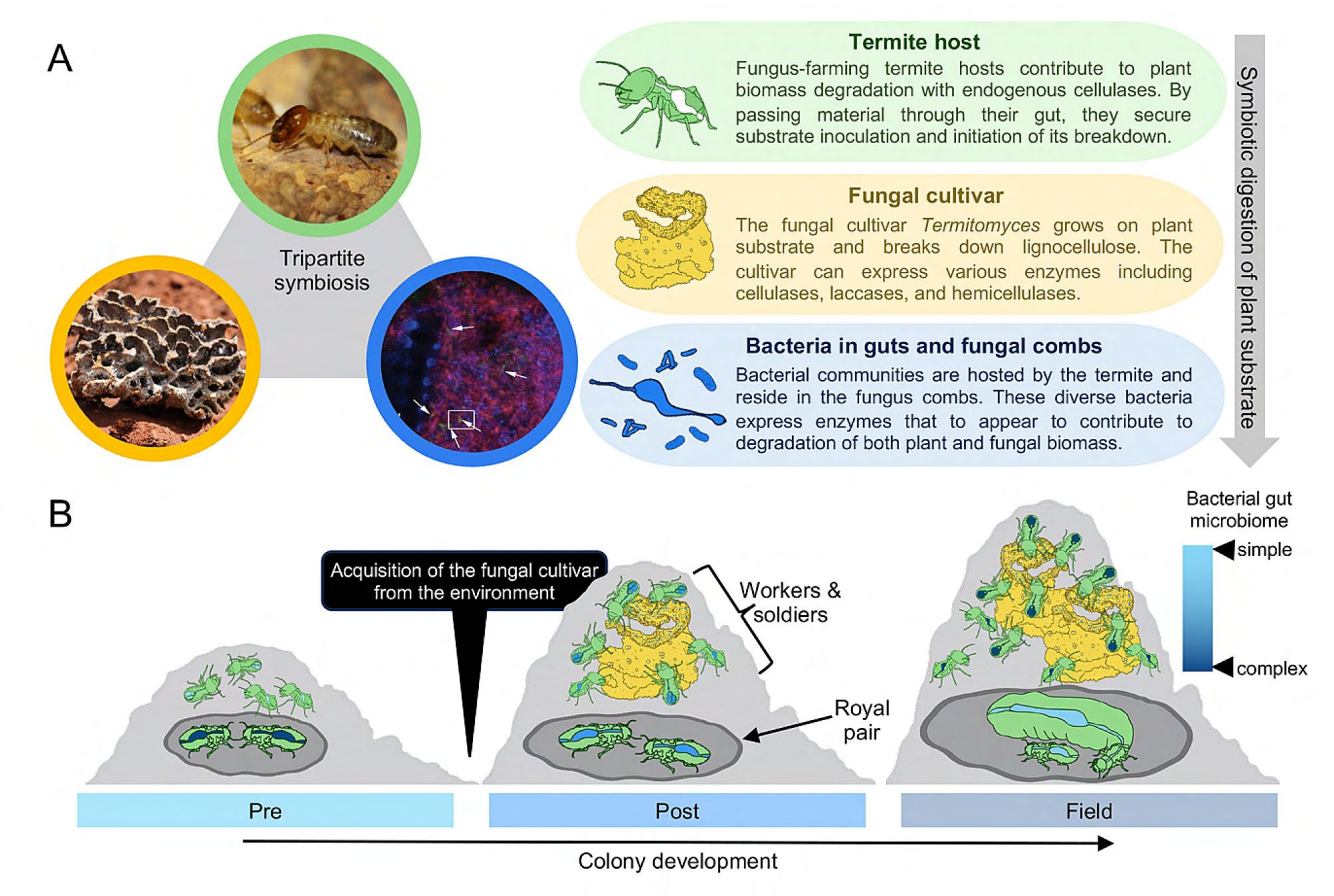
The tripartite fungus-farming symbiosis is complete after environmental acquisition of the fungal cultivar. **A**: Schematic of the tripartite symbiosis and the contributions from the termite host and symbiotic microbial partners to digestion and degradation of plant biomass. For a detailed description of the biomass degradation process, see [27]. Photos used with permission from Saria Otani. **B**: Assembly of the symbiosis over colony development and experimental timepoints. Laboratory colonies with inherited bacteria present in the guts of the royal pairs and workers prior to fungus acquisition (Pre-fungus), laboratory colonies once the fungus has been acquired (Post-fungus), and mature field colonies (Field). The gradual darkening of the gut illustrates the maturation of the gut microbiome with time, where the worker microbiome increases and the royal pair microbiome decreases in complexity

Fungiculture evolved once in termites, in the sub-family Macrotermitinae [17, 18]. The symbiosis involves the obligate cultivation of *Termitomyces* (Agaricales: Lyophyllaceae) fungi in gardens (fungal combs) [17–20] built from foraged plant material and asexual fungal spores mixed during a first termite gut passage [20, 21]. The *Termitomyces* cultivar is horizontally acquired in most termite host species [22]. Once established, the fungus degrades plant biomass to near-completion and, in doing so, provides a nutrient-rich food source for the termites [23, 24]. The fungal genus displays considerable phylogenetic congruence with termite hosts [17, 25], implying millions of years of coevolution. *Termitomyces* exhibits some degree of complementarity in enzymes for plant degradation compared to the termites and their gut microbiota [23]. In mature colonies, both termite guts and fungus combs host communities of bacterial symbionts [15, 16], where the termite gut microbiomes reflect dietary differences and division of labour [26]. Most past work on the tripartite symbiosis has focused on contributions to plant biomass decomposition [21, 23, 27–30] (Fig. 1A). The termite host genome encodes an endogenous cellulase along with primarily oligosaccharide-targeting enzymes [23]. The fungal cultivar employs a rich set of enzymes [23, 31] and presumably also uses Fenton chemistry [31] to digest a range of plant substrates; yet, with an apparent reduced capacity for oligosaccharide breakdown [23]. These oligosaccharides appear to be utilized by the rich and specialised bacterial microbiome, dominated by Firmicutes, Bacteroidetes, Spirochaetes, Proteobacteria, and Synergistetes [15, 26] that hold genes coding for primarily oligosaccharide metabolism [23]. The limited role in initial digestion of plant-derived components and the enrichment of enzymes for fungal degradation make the fungus-farming termite gut microbiome substantially different from those of other termites [23, 29, 32].

The assembly of the fungus-farming termite symbiosis is a multi-stage process initiated when the royal pair (the queen and king) start the colony (Fig. 1B). The royal pair carries with them a diverse and non-random set of gut bacterial symbionts from their colonies-of-origin [33, 34]. A substantial portion of this microbiome is transmitted to the first worker termites, making up almost half of their gut bacterial diversity [33]. This implies that at the incipient colony stage, both the fungal cultivar and a portion of the termite gut microbiome are – for most termite species – yet to be recruited to the symbioses. At this stage, the royal pair presumably lives on energetic reserves of body fat and wing muscles to produce the first cohort of workers [24, 35], to whom they reliably transfer gut microbes. After this the royal microbiome gradually depletes in both diversity and load [33, 34]. Workers forage plant material to form a primordial comb that serves as their nutrient source [24] and it is eventually inoculated by sexual spores of *Termitomyces* [20]. Once this primordial comb develops into fungus comb, the tripartite symbiosis is fully established, allowing efficient degradation of plant material [24, 30]. The functional contributions of the termite gut microbiome in mature colonies to digestion is quite well-understood. However, it remains unknown how gut microbiomes sustain incipient colony functions prior to cultivar acquisition and if bacterial functions are modulated by fungus comb establishment.

To test this, we determined the composition of gut bacterial communities and predicted their potential functional contributions before and after fungus acquisition (Fig. 1B). We sampled colonies of the fungus-farming termite *Macrotermes natalensis* at three timepoints during development: laboratory-reared colonies before the acquisition of the fungus (Pre-fungus), laboratory-reared colonies after introduction and establishment of the fungus (Post-fungus), and mature field colonies (Field). We hypothesised that either the inherited gut microbiota (Pre-fungus) would contain largely all microbial functions needed to sustain mature colonies, or that recruitment of bacterial taxa (and hence functions) would occur after fungus garden establishment and drive the mycolytic nature of gut microbiomes [32]. We established the composition and function of the gut microbiome with metabarcoding of bacterial communities, shotgun metagenomics, and enzyme assays. As metagenome sequencing depth was insufficient to accomplish binning of bacterial sequence reads into metagenome-assembled genomes, we focused on two representative and critical microbiome functions: carbohydrate-active enzymes (CAZymes) and nitrogen cycling genes.

## Materials and methods

### Colony collection and experimental design

For metagenomic characterization of the microbiome, colonies originating from Pretoria, South Africa, were collected and established in 2016 by [24]. Pre-fungus laboratory colonies were created by crossing alates from three maternal field colonies (that we call red, green and blue) with one paternal field colony [24]. Pre-fungus colonies were sampled at three months old, before the fungus comb was introduced (red *n* = 3, green *n* = 4, blue *n* = 4). At all timepoints, we collected large (major) and small (minor) worker and soldier termites. At this time, the colonies did not exhibit foraging tunnels above ground and were not fed any plant substrate; thus, any organic material the termites consumed originated from the soil surrounding the colony. Subsequently, the remaining colonies were provided wild oats, and at 3.5 months of age were inoculated with fungus comb with nodules, containing asexual spores, from a single field colony to establish the comb. At 4.5 months the colonies were provided wood in addition to hay and oats. Post-fungus colonies were then sampled at 9 months of age (red *n* = 3, green *n* = 3, blue *n* = 3), and they had thus been with their fungal cultivar for 5.5 months. Along with the termites, we also collected fungus comb. Field colony samples were from the maternal colonies that crosses originated from Both fungus comb and termites were stored in -80 °C until further processing and DNA extraction.

For bacterial metabarcoding and enzyme assays of the gut microbiome, colonies were collected and established in 2018 [24]. Pre- and Post-fungus colonies represented four maternal colonies crossed with a single paternal colony, where the maternal colonies were different from those used in 2016 but from the same location. All other treatment and sampling of the laboratory colonies was done as in 2016. Nineteen Pre-fungus colonies from four maternal lineages were used for metabarcoding (red *n* = 4, blue *n* = 4, green *n* = 5, yellow *n* = 6) and 12 were used for enzyme assays (red *n* = 3, blue *n* = 3, green *n* = 3, yellow *n* = 3). Similarly, 20 Post-fungus colonies were sampled for metabarcoding (red *n* = 4, blue *n* = 4, green *n* = 6, yellow *n* = 6) and 12 for enzyme assays (red *n* = 3, blue *n* = 3, green *n* = 3, yellow *n* = 3). Four Field colonies were used for metabarcoding and enzyme assays (Table S1).

### DNA extraction, sequencing, and droplet digital PCR

We performed droplet digital PCR (ddPCR; Bio-Rad, Denmark) to assess the absolute bacterial load in each sample sent for 16 S *rRNA* metabarcoding, using the number of gene copies as a proxy for bacterial load. ddPCR was performed with primers 63F and 355R targeting the 16 S *rRNA* V2 region, as previously used to quantify bacterial loads in termites [33] and ants [36]. In brief, reactions and quantification were performed according to manufactures instructions using QX200 EvaGreen Supermix (Bio-Rad, Denmark). Each reaction contained 11 ml 2x EvaGreen Supermix, 9.2 mL molecular water, 0.4 ml of 10 mM forward primer, 0.4 mL of 10 mM reverse primer, and 1 mL of sample. Of these 22 mL, only 20 mL was used in droplet generation. The following ddPCR protocol was used: first annealing 5 min at 96 °C, 40 cycles of denature 30 s at 95 °C and annealing/extension 1 min at 55 °C, signal stabilisation 5 min at 4 °C and 5 min at 90 °C, all at a ramp rate at 2 °C per second. Initial tests indicated that all termite samples and Post-fungus fungal comb samples had high numbers of 16 S *rRNA* gene copies, and they were hence diluted 100-fold, while Field fungal comb samples were not. These dilutions allowed for clear separation of positive and negative droplets to accurately quantify 16 S *rRNA* gene copy numbers. Negative extraction controls and non-template controls were included in ddPCR runs. Unlike conventional qPCR, ddPCR does not require technical replicates because an estimated error rate is provided per sample. Eight samples were therefore re-run to assure that samples fell within this error rate. The 16 S *rRNA* copy number per termite gut was then back-calculated based on dilutions and the number of guts per extraction.

Metabarcoding of bacterial communities was performed on Pre-fungus, Post-fungus, and Field colonies. Major workers and soldiers were by rinsed in 70% ethanol and then in molecular dH_2_O to reduce surface contaminants, after which guts were dissected aseptically. Four to six workers were pooled per Pre-fungus (*n* = 19) and Post-fungus (*n* = 20) colony, to represent a sample each, and four samples were included per Field colony (*n* = 16). Soldiers from each lineage for Pre- and Post-fungus timepoints (*n* = 4) were also sampled, and six soldiers were pooled across three Pre-fungus and three Post-fungus colonies for each sample due to the small colony sizes. One soldier sample was also allocated for each of the Field colonies (*n* = 4). Further, approximately 1 cm^3^ of the fungus comb from each Post-fungus colony and Field colonies were aseptically sampled, considering fungal and gut microbiomes share bacterial species [16]. Lastly, four negative controls were included. DNA was extracted using the DNeasy PowerSoil Pro Kit (Qiagen, Denmark) according to the manufacturer’s instructions. Two samples from each of three Field colonies (*n* = 6) were extracted with the protocol used for the metagenomic samples (see below) to test for extraction bias. DNA samples were sent to BGI (Shenzhen, China) for library construction and amplicon sequencing of the 16 S *rRNA* V3-V4 region using the primers 341 F and 806R. Libraries were created with PCR amplification of 30 ng of template DNA with 16 S *rRNA* fusion primers, followed by purification with Agencourt AMPure XP beads and tagging. Library size and concentration were determined with an Agilent 2100 Bioanalyzer, and sequencing was done on a HiSeq platform to a minimum depth of 40,000 reads per sample.

Metagenomic sequencing was conducted on Pre-fungus, Post-fungus, and Field colonies. First, major and minor workers were dissected as done for the metabarcoding samples. To acquire adequate biomass for extraction and sequencing, 7–10 guts were pooled per sample. Major and minor castes were pooled because their taxonomic composition has been found to be comparable [26]. One sample of pooled guts was extracted from each Pre-fungus (*n* = 11) and Post-fungus (*n* = 9) colony as described above, and 1 to 2 samples were extracted per Field colony (*n* = 5). DNA was extracted using a modified DNeasy Blood and Tissue Kit protocol (Qiagen, Denmark) [32]. Library preparation, sequencing, and initial quality control was conducted at BGI (Shenzhen, China). In brief, DNA was sheared to 300 bp, and overhangs from fragmentation were repaired with T4 DNA polymerase, Klenow fragment, and T4 polynucleotide kinase. Then 3’ ends were A-tailed and ligated with paired-end adaptors. Fragments were purified with gel electrophoresis and selectively enriched, amplified, and indexed with PCR. The quantified libraries were then sequenced with 100-bp lengths using DNBseq, acquiring a minimum of 15 GB of data per sample (see Table S2 for read counts).

### Enzyme assays of termite guts

Enzyme assays of termite worker and soldier guts were performed for Pre-fungus, Post-fungus, and Field colonies utilizing the azurine-crosslinked (AZCL) substrates Xylan, Galactan, Curdlan, Amylose, Arabinan, Arabinoxylan, Galactomannan, HE-cellulose, and Xyloglucan (Table S3; Megazyme, Ireland) (Table S3). Extracts were made for Pre-fungus (*n* = 12), Post-fungus (*n* = 12), and Field (*n* = 12) colonies. Crude enzyme extractions were performed as described by [30]. In brief, termites were thawed on ice and guts were dissected aseptically in sterile dH_2_O. A suspension of 100 mg gut per mL sterile dH_2_O was created by crushing the tissue with a sterile pestle and vortexing. The solution was centrifuged at 15,000 g for 15 min at 4 °C, and the protein supernatant was removed.

Enzyme extracts were applied to plates containing AZCL substrates to measure the relative enzymatic activity. AZCL substrates are purified polysaccharides or proteins containing azurine dye crosslinks. When enzymes cleave the specified linkage in a substrate, the dye fragments are released and form a halo. Plates were made using a buffer of 23 mM phosphoric acid, 23 mM acetic acid, and 23 mM boric acid [30, 37], and adjusted to pH 7, which is the average pH for a *Macrotermes* gut [38]. The remaining plate preparation followed the manufacturer’s instructions, including a standardized well size in plates [37]. Immediately after centrifugation, 15 mL enzyme extract was applied to the plates in duplicate or triplicate technical replicates. A negative control was also used for each substrate, in which a gut extract was heated at 95 °C for 20 min to denature any enzymes, after which the extract was applied to the plate. Plates were incubated at 28 °C for 24 h, and then photographed under standardized conditions with a light and a scale bar. Halo sizes were measured in ImageJ [39], and averaged for technical replicates.

### Metabarcoding analysis

Analysis of metabarcoding data was first performed in R (v 4.1.2) [40] to determine amplicon sequence variants (ASVs), and subsequent taxonomic assignment and data exploration were performed in R (v.4.3.1) [40]. Two samples (SampleID 99, SampleID 100) were removed due to low sequencing quality. We employed the dada2 pipeline (v 1.22.0) [41] to filter, trim and merge paired-end reads. The default parameters were used, with the following adjustments in *filterAndTrim*: *truncLen* set to c(270, 260) and *maxEE* set to c(2,4); in *mergePairs*: *minOverlap* set to 20. Non-target length sequences were removed from the sequencing table (8 total), and chimeras were removed, leaving 8,922 ASVs and 93% reads remaining. Taxonomy was assigned with dada2 *assignTaxonomy* using the preformatted *silva_nr99_v138.1_train_set.fa.gz* database. ASVs assigned to chloroplasts and mitochondria were removed as were ASVs found in only one sample or with < 100 reads across all samples. Next, we identified and removed contaminants using the prevalence method of the R decontam package v1.18.0 [42]. The resultant feature table was standardized by square rooting with base R *sqrt*, and then normalized with Wisconsin double standardization with the vegan package [43] v2.6.4 *wisconsin* function.

### Metagenome quality filtering, assembly, and annotation

Raw reads were cleaned and filtered (Phred score > 8) using *bbduk.sh* from the BBtools package (BBMap) v38.89. To remove host-derived sequences, paired-end reads were mapped to the termite genome *Macrotermes natalensis* v.1.0 [23], and the fungal cultivar genome *Termitomyces* using the Burrows-Wheeler Aligner (BWA) tool [44] v.0.7.16a (*bwa mem*) with default settings. The de-hosted reads (Table S4) were extracted with samtools (*–f 4*). The resulting paired-end fastq files were reordered and singletons discarded with repair.sh (*repair = t*) from the BBtools package. Two samples were removed (16 and 47b) due to a 10-fold difference in the number of reads compared to other experimental samples, after removal of host sequences (Table S4).

Metagenomes were co-assembled with metaSPADES v.3.15.5 [45] with default settings using kmers (-*k*) of 21, 33, 55, 77. Co-assemblies were performed according to matriline and timepoint, apart from the Pre-fungus timepoint where all samples were co-assembled together, as microbial load was low and resulted in fewer bacterial reads recovered (Table S4). Contigs below 1000 bp were removed using *reformat.sh* from the BBtools package. Samples were next inferred by mapping reads to the co-assembly they helped create on a by sample basis with BWA [44] v. 0.7.16a (*bwa mem*). Read recruitment to each contig was determined with Anvi’o *anvi-profile-blitz* [46] taking the mean coverage of reads from a given sample to a given contig. This was used to estimate relative abundance of a contig in a sample. Metagenome annotation was performed with Prodigal v.2.6.3 on default settings in metagenome mode (*-p meta*) [47, 48].

### CAZyme and nitrogen cycling gene predictions

CAZymes [49] were recovered from the matriline co-assemblies of bacterial communities, *M. natalensis* termite and *Termitomyces* fungal host genomes [23], with the dbCAN4 [50] v4.0 pipeline. This pipeline utilizes a HMMer search based on two curated HMM profiles alongside a DIAMOND [51] search against the pre-annotated CAZyme nucleotide database [52]. Subsequently, predicted CAZyme families were determined by taking the family shown to have the highest number of conserved peptides for each database individually. Only instances where two out of the three databases identified the same CAZyme family were kept. Where all three databases identified a CAZyme, the prediction from two out of three databases was chosen. Genes involved in nitrogen cycling were recovered from the metagenome assemblies and host genomes with the NCyc tool [53] using DIAMOND v.2.0.6 [51] to search against the curated NCycDB database. Relative abundance of a CAZyme or nitrogen cycling gene was assumed to be as the relative abundance of the contig on which the gene was found.

### Alpha diversity indices and enzyme activity analyses

All analyses were performed using R v4.2.2 [40] in RStudio. We calculated the Chao1 richness of 16 S *rRNA* ASVs on unnormalized data using the *estimateR* function in the vegan package [43] v2.6.4. We determined the effect of timepoint on Chao1 richness and log-transformed 16 S *rRNA* gene copy counts via ANOVAs using *aov* and *TukeyHSD* from base R (~ timepoint * sample type) and effect sizes were determined with effectsize v0.8.3 [54] *cohens_f* function.

We calculated Observed richness and Shannon diversity on the relative abundance of CAZyme families and NCyc genes using the *estimateR* and *diversity* functions in the vegan package [43]. The effect of timepoint on Observed richness and Shannon diversity was determined with base R *aov* and *TukeyHSD* functions (~ timepoint * matriline) and effect sizes were determined with effectsize [54] v0.8.3 *cohens_f* function). To establish statistical differences in enzyme activity between Pre-fungus and Post-fungus/Field timepoint, we used ANOVAs via base R *aov* and subsequent pairwise testing with *TukeyHSD* functions (~ *timepoint*). Effect sizes were determined with effectsize v0.8.3 [54] *cohens_f* function.

### Beta diversity indices

Compositional variation between timepoints for 16S *rRNA* ASVs, CAZyme families, NCyc genes and AZCL enzyme activities was determined by calculating Bray Curtis dissimilarity with *vegdist* from vegan [43] v2.6.4 (*method=”bray”*). Principle Coordinate Analysis (PCoA) ordination plots were generated using ape [55] v.5.6.1 (*pcoa*), and the first two components were subsequently visualised with ggplot2 [56] v3.4.2. To determine if timepoint affected compositions, we used Permutational Multivariate Analysis of Variance (PERMANOVA) in *adonis2* from vegan [43] v2.6.4 using the model (~ timepoint * matriline) on the distance matrix generated by Bray-Curtis dissimilarity measures. Pairwise comparisons of each timepoint were explored with *pairwise.adonis2* from the pairwiseAdonis package v4 (https://github.com/pmartinezarbizu/pairwiseAdonis) using the same model.

## Results

### The microbial load and alpha diversity of the gut microbiome increase after fungus acquisition

**Fig. 2:**
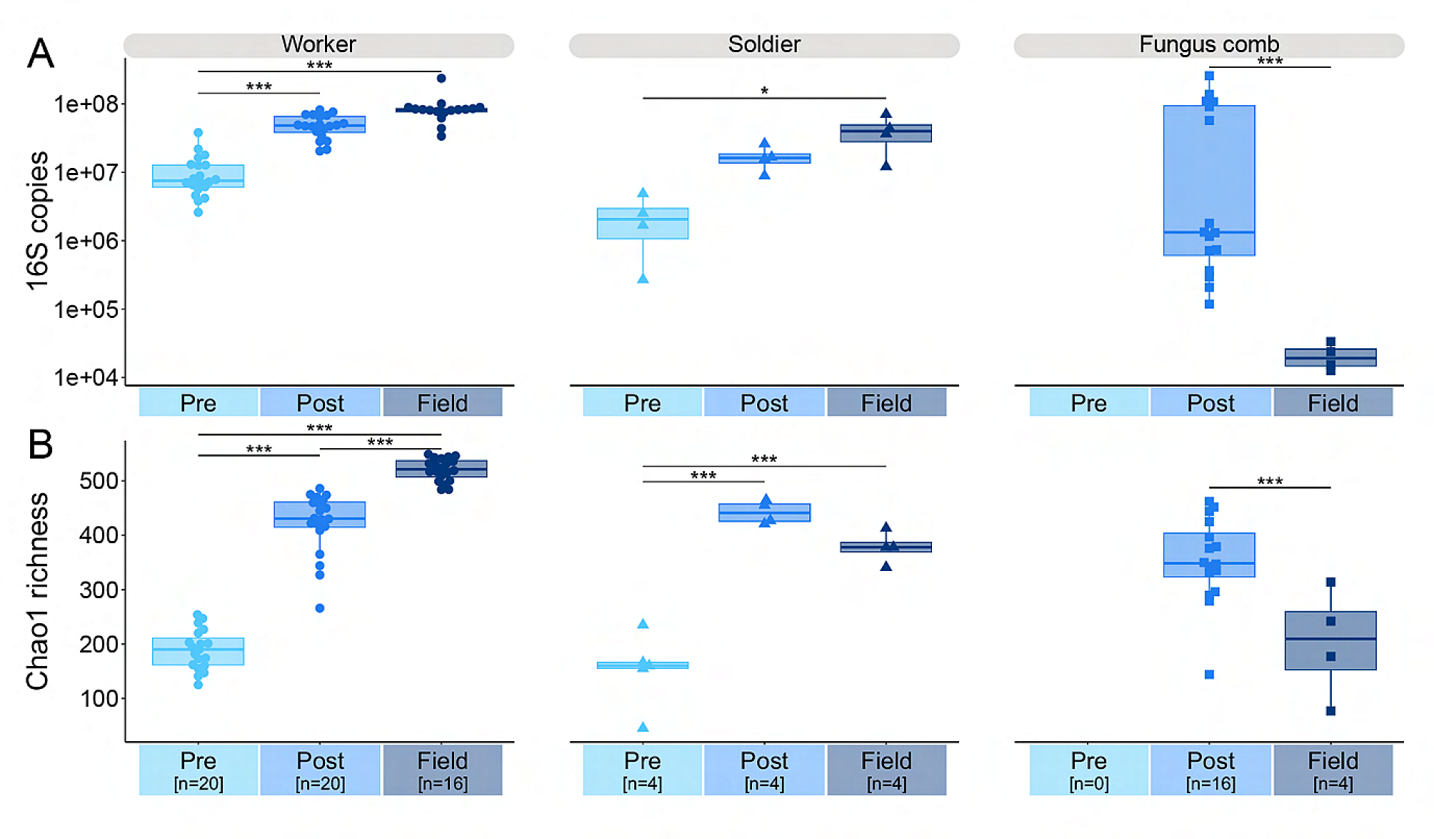
Fungus acquisition leads to an increase in gut bacterial load and diversity. **A**: Relative bacterial loads based on quantification of the 16 S *rRNA* gene using ddPCR, depicted using all data points, which indicate the number of gene copies per individual termite gut or 1 cm^3^ piece of fungus comb. **B**: Chao1 richness based on the unnormalized 16 S *rRNA* ASV data for worker and soldier gut microbiomes as well as fungal combs for the three timepoints. Sample sizes provided in brackets after timepoints on the x-axis. Standard box and whisker plots indicate the median with the central line in the box, the first and third quartiles with the box, and the minimum and maximum of the upper and lower quartiles with the whiskers. Horizontal lines indicate statistically significant comparisons from TukeyHSD post-hoc comparisons and asterisks indicate significance levels: *p < 0.05 and ***: *p* < 0.001 (full results in Table S5)

Bacterial load, quantified with ddPCR of 16 S *rRNA* gene copies, was significantly affected by timepoint (ANOVA: F_2,80_ = 4.632, *p* = 0.013, Cohen’s F = 0.34), and furthermore depended on sample type, as evident from the significant interaction (F_3,80_ = 17.79, *p* < 0.001, Cohen’s F = 0.82). Pairwise examination indicated that while bacterial load was indistinguishable in workers and soldiers between Post-fungus and Field colonies, both timepoints had significantly higher loads than Pre-fungus colonies (Fig. 2A; Table S5). In contrast, while colony maturity in general increased bacterial load in termite guts, it decreased the load of fungus combs (Fig. 2A; Table S5). Variation was larger between fungus comb samples at the Post-fungus timepoint, potentially due to variation in the proportion of comb that included fresh gut deposits. The low and consistent loads in Field combs imply that bacteria are not prevalent in combs of mature colonies (Fig. 2A).

Timepoint had a significant effect on Chao1 richness (ANOVA: F_2,84_ = 185.7, *p* < 0.001, Cohen’s F = 2.10) that was dependent on sample type (F_2,84_ = 19.75, *p* < 0.001, Cohen’s F = 0.84). Richness increased across all timepoints in workers, but only between Pre-fungus and Post-fungus/Field in soldiers (Fig. 2B; Table S5). The similarities between Post-fungus and Field suggest that laboratory conditions for Pre- and Post-fungus colonies had only a minor, if any, impact on bacterial loads and richness. As was the case for bacterial load, Chao1 richness decreased from Post-fungus to Field fungus combs (diff = 150.723, *p* < 0.001) (Fig. 2B). Fungus combs from Field colonies were less rich than Post-fungus colonies and seemingly more variable, albeit with a small sample size (Fig. 2B).

**Fig. 3:**
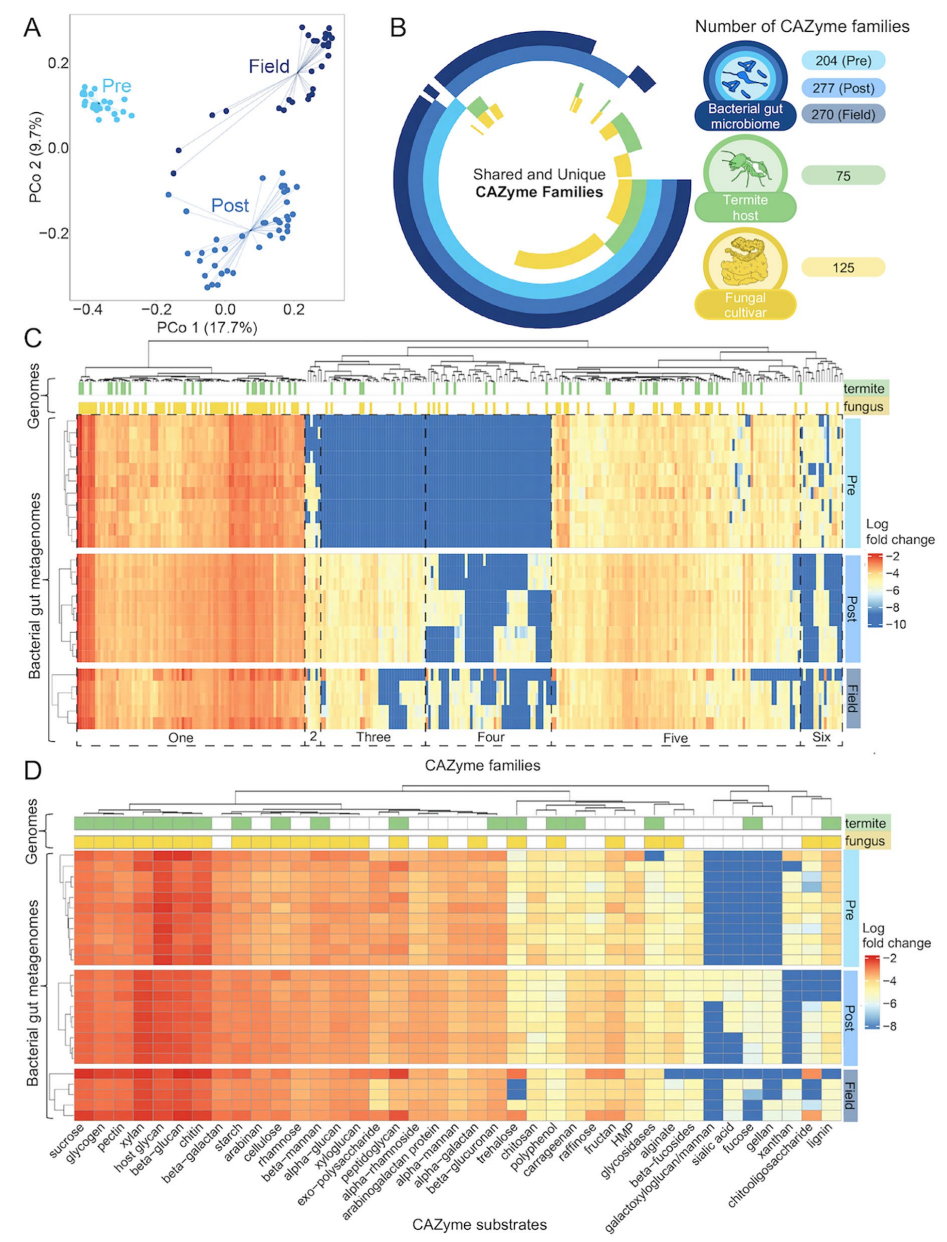
Shifts in carbohydrate active enzyme (CAZyme) potential of the fungus-farming termite gut microbiome through development. **A**: PCoA of the beta diversity of the termite gut bacterial microbiomes across timepoints in colony development, based on Bray-Curtis distances from 16 S *rRNA* amplicon sequencing. **B**: Complementarity in CAZyme families identified in bacterial gut metagenomes, along with the termite host and fungal cultivar genomes. While some CAZyme families are shared across symbiotic partners, others are unique. Additional CAZyme families are acquired by the bacterial gut microbiome after fungus acquisition, changing from 204 CAZyme families in the bacterial gut microbiome of Pre-fungus workers to 277 and 270 in Post-fungus and Field worker bacterial gut microbiomes, respectively. **C**: The log relative abundance of CAZyme families encoded for by termite gut bacteria, grouped visually by CAZyme family group on the horizontal axis (Groups one to six) and clustered by timepoints on the vertical axis. Clustering is based on the complete method of hierarchical clustering using Euclidian distances. CAZyme families found in the genomes of the termite host *M. natalensis* (termite) and *Termitomyces* cultivar (fungus) are indicated at the top of the heat map. **D**: The putative functional capacity of termite gut bacteria, as predicted by substrates targeted by the identified CAZyme genes, displayed as cumulative log relative abundances. Clustering method, heatmap scale, and indications of the putative capacity of termite and fungus to metabolise the substrates are based on their respective genomes, depicted as in **C**

Multivariate analysis of the metabarcoding data using PERMANOVA allowed us to infer that 24.2% of the variation in community composition could be explained by timepoint (F_2,84_ = 16.84, R^2^ = 0.2427, *p* < 0.001) (Fig. 3A). Pairwise comparisons further demonstrated that all timepoints differed from each other but that Post-fungus and Field were more similar (Table S5).

### Metabolic potential changes associated with the acquisition of the fungal cultivar

To infer the metabolic potential of the fungus-farming termite gut microbiome with regard to carbohydrate metabolism, we investigated the catalogue of enzymes involved in the breakdown of complex carbohydrates from the CAZy database using dbCAN4 (v.4.0.0). We identified a total of 292 unique CAZyme families across timepoints and symbiotic partners. The number of CAZyme families in the gut microbiome increased by approximately 1/3 with the acquisition of the fungal cultivar, from 204 (Pre-fungus) to 277 (Post-fungus), or 270 (Field). This clarified that fungus acquisition did alter the potential function of the microbiome, although most CAZyme families were present at colony foundation (Fig. 3B). The expansion in the number of CAZyme families in the gut microbiome with the acquisition of the fungal cultivar introduced additional putative functions that are absent in the termite host and fungus genomes. Complementarity observed between termite, fungal, and bacterial CAZyme contributions is consistent with previous findings [23], and there was also overlap in carbohydrate metabolism between symbiotic partners. The fungus and termite shared respectively 35.3% and 18.5% of CAZyme families identified in microbiomes across time points.

Given the clear expansion in a portion of the carbohydrate active functions of the gut microbiome, we further examined the relative abundances of CAZyme families. Heatmaps of the logged relative abundance profiles of CAZyme families revealed six distinct groupings by timepoint (Fig. 3C). Families in Group one were present at all timepoints in relatively high abundance. Group two was variably present in Pre-fungus but always present in Post-fungus and Field colonies, while group three was absent from Pre-fungus but almost always present in Post-fungus and Field. Group four was also absent from Pre-fungus but only variably present in Post-fungus and Field, suggesting functional maturation of metabolic potential in the microbiome after fungus acquisition. Group five families were, as Group one, consistently present across timepoints but in lower relative abundances, and finally Group six was always present in Pre-fungus but only variably present in Post-fungus and Field, potentially indicating loss of function during microbiome maturation. Therefore, a core set of functions appear to be maintained through colony development, making up the majority of carbohydrate metabolism in the gut, while horizontal transmission of the fungus drives a general increase in the abundance of other putative functions.

The clear groupings of families based on relative abundances were as a whole consistent with functional shifts indicated by substrate predictions from dbCAN4 (Fig. 3D). Functional capacity stays consistent across timepoints with variation primarily in enzymes targeting less-relevant substrates. We saw a lack of enzymes putatively targeting sialic acid, fructose, and gellan in the Pre-fungus timepoint, while Post-fungus and Field variably lacked enzymes targeting xanthan and chitooligosaccharides (Fig. 3D). Notably, enzymes targeting lignin were significantly reduced in relative abundance from Pre- to Post-fungal acquisition (for pairwise comparisons, see Table S5). The absence of predicted substrates for many of the CAZymes meant that we could only establish a partial picture of how acquisition of the fungal cultivar affects substrate targets. However, it was evident that enzymes targeting the majority of plant (e.g., cellulose, pectin, hemicellulose, and lignin) and fungal (e.g., α-glucan, β-glucan, chitin, and mannan) cell wall components were abundant at all timepoints suggesting ample ability to aid in their breakdown from colony inception.

We then compared Shannon diversity and Observed richness of CAZyme potential across timepoints, revealing that time had a significant effect on both CAZyme diversity and richness with Pre-fungus being substantially lower for both (ANOVA – Shannon: F_2 − 16_ = 76.51, Cohen’s F = 3.09, *p* < 0.001; Observed: F_2 − 16_ = 1281, Cohen’s F = 12.66, *p* < 0.001) (Fig. S1A). Matriline also had a significant effect on Observed richness (F_2 − 16_ = 79.34, Cohen’s F = 3.15, *p* < 0.001) but not Shannon diversity (F_2 − 16_ = 1.660, *p* = 0.221). Pairwise TukeyHSD comparisons showed Pre-fungus to be significantly lower than both Post-fungus and Field, while these were significantly different for Observed richness but not Shannon diversity (Fig. S1, Table S6). This suggests that acquisition of the fungus not only shifts the microbiome to host a greater diversity of bacteria, as was also evident from the ASV analysis, but also encoded CAZymes.

**Fig. 4:**
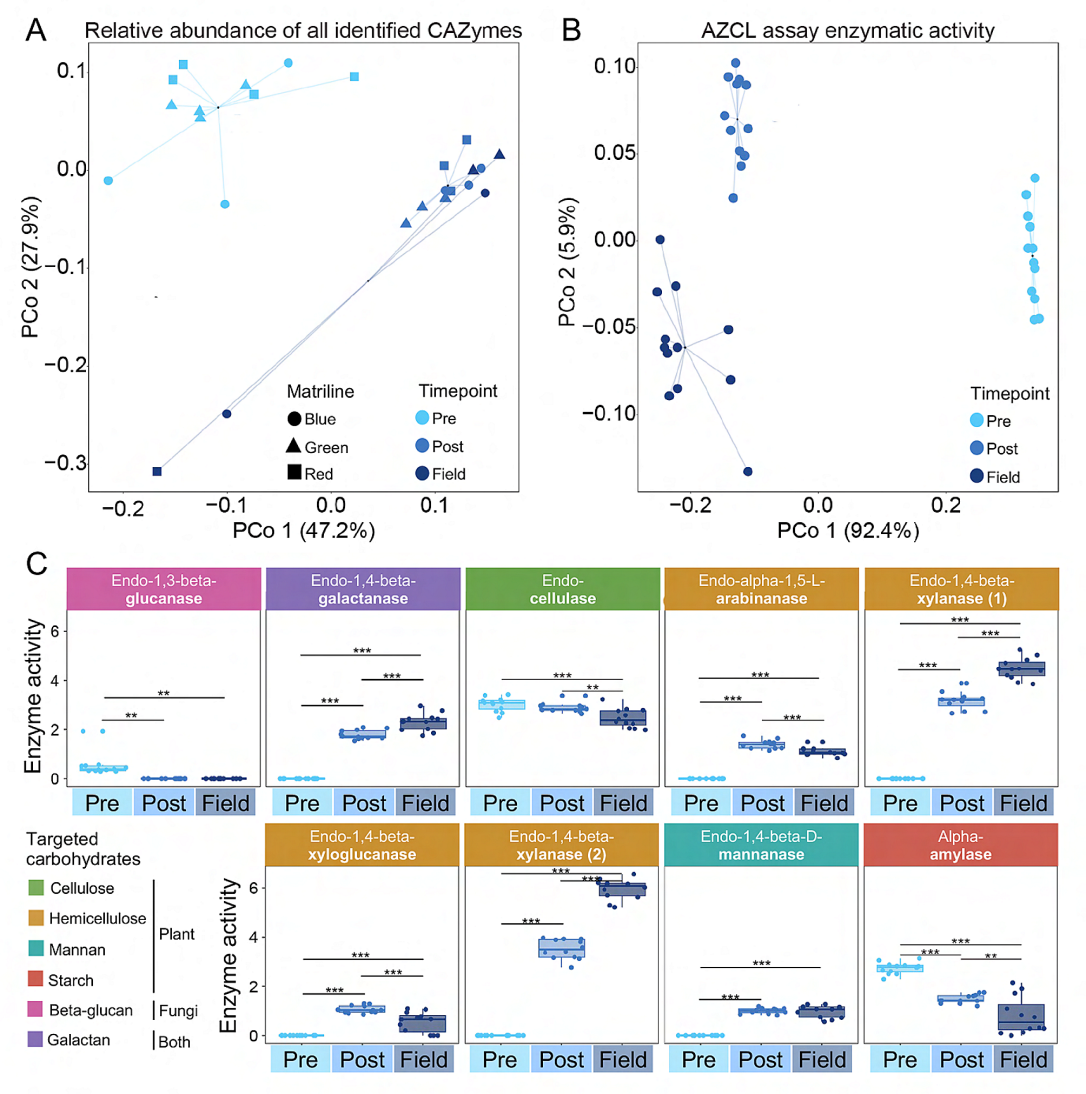
Similarities in profiles of all identified CAZyme family relative abundances and AZCL enzyme activities. **A**: PCoA based on Bray-Curtis distances of CAZyme family compositions across timepoints (Pre-fungus in light blue, Post-fungus in blue, and Field colonies in dark blue). Different shapes indicate the three matriline colonies samples originated from (blue, green, and red). **B**: PCoA based on Bray-Curtis distances in AZCL enzymatic activity compositions by timepoint and matriline (labels as in **A**). **C**: CAZyme enzyme activity in termite worker guts based on selected AZCL enzyme assay results (for the full results, see Table S9). Endo-1,4-beta-xylanase is listed twice because the enzyme was tested with AZCL assays for both xylan (1) and arabinoxylan (2). Target substrates are indicated in different colours, and dots represent individual samples (*n* = 12 for all timepoints). Enzyme activities represent the relative expression in the entire gut; thus, including active termite host, bacterial, and residual fungal cultivar enzymes. Standard box and whisker plots indicate the median with the central line in the box, the first and third quartiles with the box, and the minimum and maximum of the upper and lower quartiles with the whiskers. Enzyme activities were subject to ANOVA analyses, and the significance of Tukey HSD pairwise comparisons are indicated: **: *p* < 0.01 and ***: *p* < 0.001 (Table S8)

Analysing the Bray Curtis distances between CAZyme relative abundance profiles through multivariate compositional analysis revealed that timepoint had a significant effect and explained a substantial component of the observed variation (PERMANOVA: F_2,16_ = 13.20, R^2^ = 0.4677, *p* < 0.001) while matriline did not (F_2,33_ = 0.9839, *p* = 0.4039). The pairwise comparisons further revealed that Pre-fungus stood out as particularly distinct, as indicated by larger R^2^ values (Table S7). Matriline remained insignificant in all comparisons (Table S7). These clear groupings by timepoint were also evident from PCoA clustering. Here, the reduced explanatory power of time in comparisons of Pre-fungus and Field was likely due to two samples that substantially deviated from the rest of the Field samples (Fig. 4A). These two samples were consistently more abundant in multiple CAZyme families than other Field samples (Fig. 3C). The composition analysis established that the majority of the maturation in CAZyme potential takes place between Pre- and Post-fungus stages in colony life, but likely continues until colonies reach maturity. The presence of CAZymes at similar relative abundances across timepoints thus suggests metabolic dormancy until the fungal cultivar is acquired as gut functions change with the shift in termite host diet.

### Enzymatic activities of termite guts shift with fungus acquisition

Relative abundances of CAZymes can inform the potential metabolic profiles of an environment but are not always reflective of actual enzymatic activity. Therefore, we profiled the enzymatic activity of termite guts for eight key enzymes, which metabolise plant and fungal components, through AZCL enzymatic activity assays. Multivariate compositional analysis of Bray Curtis distances between enzyme activity profiles demonstrated that timepoint explained almost all of the observed variation (PERMANOVA: F_2,33_ = 383.7, R^2^ = 0.9588, *p* < 0.001). Similar to CAZyme family profiles, pairwise comparisons showed that timepoints were significantly different from each other, and that Pre-fungus again stood out as most different (Fig. 4B; Table S5). The extreme explanatory power of timepoint was also evident from PCoA clustering into three distinct groups with little dispersion (Fig. 4B). As for the predicted CAZyme relative abundances, this indicates that the major enzyme activity differences are between Pre- and Post-fungus stages of colony life but that activity continues to change until colony maturity.

The extreme variation in enzyme activities between Pre-fungus and both Post-fungus and Field appeared to be primarily driven by enzyme activities being absent or very low prior to fungus acquisition (Fig. 4C). Timepoint had a significant effect on the AZCL enzyme activity levels (Table S8). Tukey HSD pairwise comparisons revealed that the activity of some enzymes targeting plant substrates increased with colony development, while others decreased (see Table S8 for all pairwise comparisons). For example, the activity of enzymes that act on hemicellulose and mannan increase, but those on cellulose and starch decrease. Similar activity patterns were observed in enzymes targeting fungal beta-glucans and galactans. Since genes encoding these CAZymes were consistently present in the metagenome, this is conceivably due to metabolic dormancy of the gut microbiota at this point in time. However, given that the AZCL assays were conducted on whole termite guts, some of the activity is likely also attributable to endogenous termite enzymes and fungal enzymes derived from the ingestion of fungus comb.

### The termite gut microbiome encodes a diverse range of nitrogen cycling genes

**Fig. 5:**
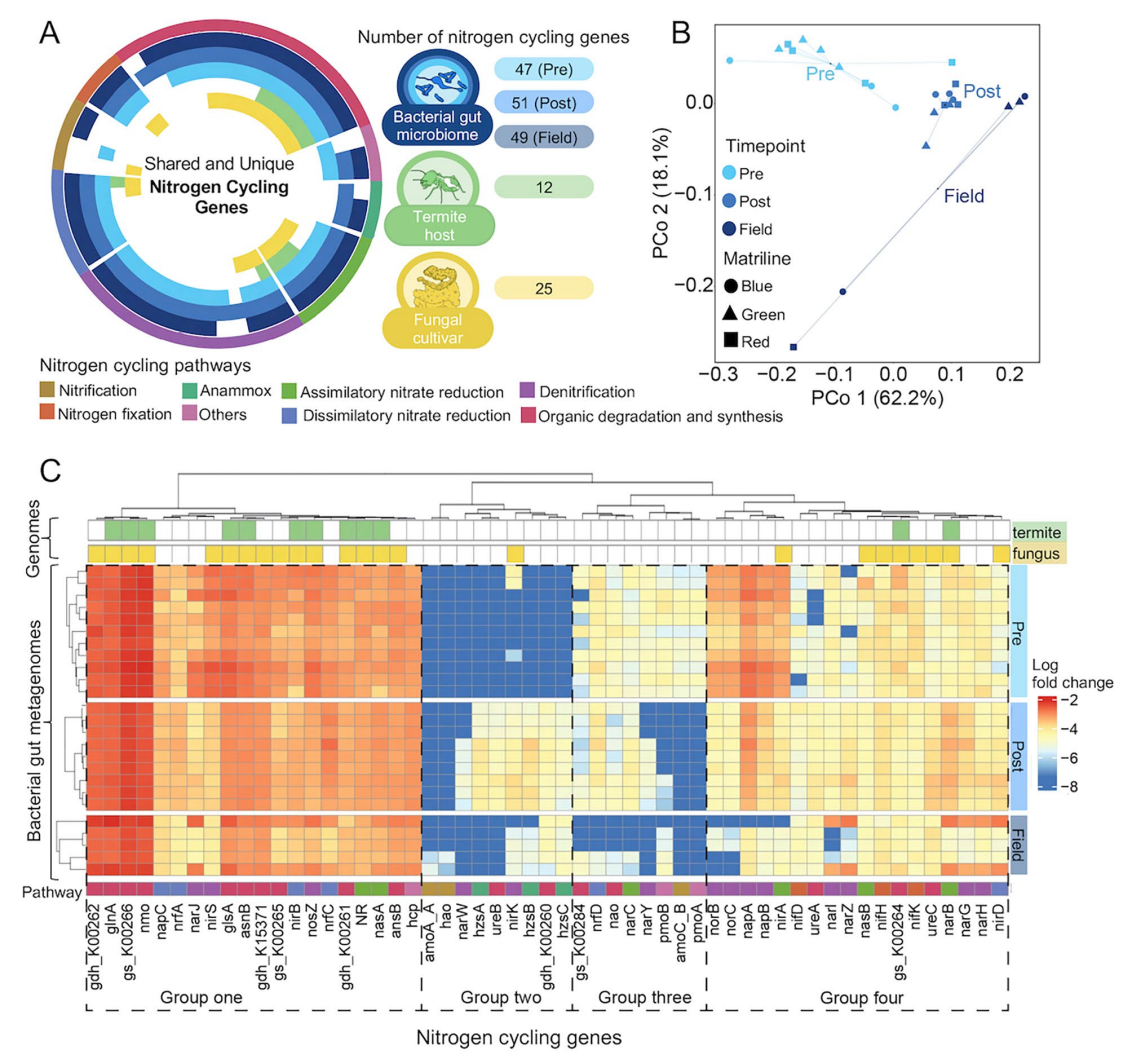
Putative nitrogen cycling contributions from the gut microbiome remain relatively consistent over colony development. **A**: Complementarity in nitrogen cycling genes (NCyc) in bacterial gut microbiome, termite host, and fungal cultivar. The genes are organised by pathway, and the number of Ncyc genes identified for bacteria, fungus and termite are given next to their respective symbols. **B**: PCoA using Bray-Curtis distance of nitrogen cycling gene relative abundances in the gut microbiome across timepoints. Shapes indicate the three different matriline colonies samples originate from (blue, green, and red) and timepoints are indicated by different shades of blue. **C**: Clustering of log fold change of putative NCyc genes encoded for by termite gut bacteria, grouped visually into four groups on the horizontal axis based on differences across time points (Groups one to four), and clustered by timepoint on the vertical axis using the complete method of hierarchical clustering with Euclidian distances. Ncyc genes found in the genomes of the termite host *M. natalensis* (termite) and the fungal cultivar *Termitomyces* (fungus) are indicated at the top of the heat map

To explore the nitrogen cycling potential of the fungus-farming termite gut microbiomes, we investigated genes found in the bacterial gut microbiome, termite host, and fungus. We identified 55 unique genes from all seven families categorised in NCycDB. Complementarity across members of the symbiosis was high but the gut microbiome contributed a substantial number of otherwise unavailable nitrogen cycling genes (Fig. 5A). *Termitomyces* and the termite host shared 43.6% and 21.8% of genes across timepoints, respectively, and these were mainly involved in organic degradation, organic synthesis, and assimilatory nitrate reduction (Fig. 5A).

Clustering of logged relative abundance of putative NCyc genes across colony development formed four groups (labelled Group one to four in Fig. 5C). Group one and four appeared to be the core NCyc genes consistently found in the gut microbiome, with Group one present in high abundance across all timepoints but Group four being notably less abundant (Fig. 5C). Group two was mostly present in Post-fungus and variably in Field but absent in Pre-fungus, with NCyc genes increasing in relative abundance with the acquisition of the fungal cultivar. Group three was always present in Pre-fungus but more variably present and abundant in Post-fungus and Field. Within Group three, there was a subgroup that for the most part was absent in Field. Grouping genes by broad metabolic pathways showed that most pathways were well represented across timepoints. A substantial portion of the nitrogen cycling genes was involved in organic degradation and synthesis and denitrification, with minor variation in the relative abundances of genes involved in these pathways (Fig. 5C). Notably, genes involved in nitrogen fixation were present in high abundance at all timepoints. The most variable pathway across timepoints was anaerobic ammonium oxidation (anammox), which was essentially absent before fungus acquisition. Nitrification was nearly absent across all timepoints. Thus, as for CAZy potential, the majority of nitrogen cycling-related potential was present across all timepoints, with only minor losses or gains post-fungus acquisition. This potential covered most of the nitrogen cycling pathway and notably also nitrogen fixation, for which microbial genes were present at all timepoint (Fig. 5C).

Similar to CAZyme potential, timepoint significantly affected both nitrogen cycling gene diversity and richness (ANOVA: Shannon: F_2,16_ = 13.20, Cohen’s F = 1.28, *p* < 0.001; Observed: F_2,16_ = 182.08, Cohen’s F = 4.77, *p* < 0.001) (Fig. S1B). Shannon diversity was consistent across matrilines, but richness was not (Shannon: F_2,16_ = 0.055, *p* = 0.6469; Observed: F_2,16_ = 17.07, Cohen’s F = 2.11, *p* < 0.001). Pairwise comparisons of Shannon diversity with TukeyHSD demonstrated Field to be lower than both Pre- and Post-fungus, while Post-fungus had the highest Observed richness followed by Pre-fungus and then Field (Fig. S1, Table S6).

The multivariate PERMANOVA further revealed that timepoint explained more than 40% of the variation in nitrogen cycling gene compositions. Timepoint significantly affected profiles (PERMANOVA: F_2,16_ = 10.65, R^2^ = 0.4320, *p* < 0.001), while compositions were consistent across matrilines (F_2,16_ = 0.6165, *p* = 0.6982) and subsequent pairwise comparisons showed that timepoints were indeed significantly different from each other (Table S5). This was also reflected in PCoA clustering (Fig. 5C), again with the two distinctly different samples (9b and 15b) having higher abundances of several genes (Fig. 5B). Maternal lineage remained insignificant across comparisons (*p* > 0.05) (Table S7).

## Discussion

### Inherited gut bacteria secure microbial functions that support mature termite colonies

Our exploration of fungus-farming termite symbiosis revealed that the bacterial gut microbiome is largely conserved over colony life and the assembly of the tripartite symbiosis. This allows us to accept the hypothesis that the inherited gut microbiota contains essentially all microbial functions of mature colonies, with relatively minor modifications associated with acquisition (horizontal transmission) of the primary food source, the fungal cultivar. Although the species richness of the gut microbiome increases after fungal acquisition, the vast majority of carbohydrate metabolism and nitrogen cycling genes were present already at colony foundation. This sharply contrasts many other animal host-microbiome associations, where marked shifts in offspring microbiome compositions and functions are associated with dietary shifts during development [9, 10, 13, 14].

Despite horizontal transmission of the co-evolved fungal cultivar, we observed only slight shifts in putative enzymes for plant and fungal degradation in the bacterial gut microbiome. While the cellulase in the host termite genome should help free glucose that can be directly absorbed, partially digested substrate components will pass to the termite hindgut [57]. Here, rich bacterial CAZymes, abundant across at all timepoints, have the capacity to target a range of plant cell wall (e.g., cellulose, pectin, hemicellulose, and lignin) and fungal cell wall (e.g., α-glucan, β-glucan, chitin, and mannan) components. The most striking shift in CAZyme potential after garden establishment was the reduction in enzymes that target lignin. This is consistent with the lignocellulolytic capacities of *Termitomyces* taking over this role in the symbiosis [23, 31]. Similarly, most nitrogen cycling-related potential in gut microbiomes was present across timepoints. The most notable change here was that potential for anaerobic ammonium oxidation, which only appeared after fungus comb establishment. Major caveats to the insights gleaned from our work are that we only evaluate gene predictions and base inferences on changes in relative abundances, which may not reflect in situ activities. However, the comparisons across timepoints nevertheless allow elucidation of any changes in key putative microbial functions of the co-evolved bacterial gut microbiome of the tripartite symbiosis.

### Assembling the fungus-farming termite symbiosis

The royal pair of a fungus-farming termite colony host a suite of host-specific enzymes in their genomes [23] and bring with them a diverse set of gut bacterial symbionts that are reliably passed on to the first offspring colony workers [33, 34]. Our findings indicate that this set of microbes hold an extensive metabolic potential that appears – at least to a very large extent – to cover the needs of mature colonies. Vertical transmission thus provides the required bacterial diversity with the necessary functional potential that allows efficient lignocellulose digestion, and conversion of a nitrogen-poor plant substrate into a high-nitrogen fungal food source for the termites. This supports previous assertions that core gut-specific bacteria, which cannot be acquired from the environment, must be inherited to maintain symbiont functions across termite colony generations [33, 34].

The establishment of the fungus comb was accompanied by an increase in bacterial richness and load within fungus-farming termite guts. This is likely due to increased productivity within guts but may also reflect some recruitment of environmental microbes. The role of the termite gut changes as the fungus comb is established. The guts become the site for sustaining host nutrition on plant and fungal components, and for seeding the new comb with plant substrate and fungal spores from nodules within the maturing comb [20, 21, 27]. Termite ingestion of plant biomass should be accompanied by a shift in the diversity of microbes that enter termite guts, including environmental bacteria. As fungus-farming termites are generalists in the substrates they harvest [21], this may allow microbial inputs of both transient taxa and taxa that establish, if they can tolerate the gut microenvironment and utilise available substrates. Furthermore, fungus comb establishment is also accompanied by the formation of bacterial microbiomes within combs, derived in part from termite gut deposits and taxa from the surrounding soil [16]. As the comb is ultimately consumed by the termites, this may involve ingesting bacteria that primarily reside in gardens and not necessarily in guts. Given that our explorations were based on DNA alone, we would detect them regardless of whether they are transient or established symbionts. Thus, future work to detail site-specific microbial activities should decipher precisely where bacterial contributions play out.

### Functional changes associated with the shift in termite diet

Our findings suggest that already in early colony life, the gut microbiome has the capacity to provide nutritional benefits to the termites [27]. The vast number of CAZymes in the bacterial gut microbiome were comparable across timepoints. Substantial increases were primarily observed for enzymes targeting galactoxyloglucan/mannan, sialic acid, fucose, and gellan. With the exception of an increased role in hemicellulose degradation (i.e., enzymes targeting mannan), these substrates do not reflect dietary changes termites should experience in the transition from a plant to a fungal diet. The most significant reduction with fungal acquisition was CAZymes putatively targeting lignin in guts, likely reflecting the changing role of gut bacteria when *Termitomyces* becomes the central lignin degrader [23, 27, 31]. In concert, the lignocellulolytic role of the gut microbiome shifts to the degradation of simpler plant biomass components [23, 29]. Albeit limited in scope, our enzyme assays reflected such relevant shifts in microbial activities, specifically for hemicellulose, mannan, and galactan. As the genetic potential for the enzymes is present at the Pre-fungus timepoint, the low activity early in colony life implies dormancy in hemicellulolytic enzymes prior to fungus acquisition. This underlines the need for future work to not only gain deeper insights into the functional capacities of termite gut microbes – including also Archaea [29] and yeasts [58] – in plant biomass degradation, but also establish their activities at relevant time points in colony life.

We found a strikingly similar pattern for nitrogen cycling pathways, indicating that the termite gut microbiome is capable of supporting a range of pathways in nitrogen cycling from the onset of colony foundation. Most notably, the gut microbiome contains genes for nitrogen fixation, organic degradation and synthesis, denitrification, nitrate reduction, and others. The Ncyc potential of the farming termite gut is thus vastly wider than those of the host termite and cultivar fungus, where most Ncyc genes were found in degradation and synthesis and assimilatory nitrate reduction. Interestingly, a few genes for nitrogen fixation where also discovered in *Termitomyces*, suggesting that it may contribute to generating the high nitrogen content that is observed in the fungus [59]. Certain bacterial taxa have previously been identified to contain all needed genes for nitrogen fixation (e.g., members of the order Campylobacterales and the genus *Kosakonia* [29]). Finding the full set of *nifH* genes is also consistent with previous work showing that fixation of nitrogen occurs in termite guts [60], and that an unspecified nitrogen source, most likely symbiotic nitrogen-fixing bacteria, must contribute to nitrogen supply in termite nutrition [61]. The only nitrogen-cycling potential that appeared to change from Pre- to Post-fungus conditions was anaerobic ammonium oxidation, consistent with the presence of ammonia-oxidizing bacteria in both fungus-farming and other termites [62, 63].

Our approaches indicate complementarity in genes associated with two key functions, carbohydrate and nitrogen metabolism. However, the functional analysis was based on relatively coarse sequencing information and predictions that are unlikely to capture the full symbiotic genetic repertoires. Future metagenome-assembled genomes (MAGs) of gut (and comb) bacteria will be needed to fully establish whether shared enzyme families and gene products across hosts and symbionts represent overlaps in functions. This may either represent situations where functions are expressed in a context-dependent manner by individual organisms or through complementary expression of specific steps in pathways encoded for collectively by host and symbiont genomes.

### Conclusions and perspectives

Our study is the first to examine how the assembly of the fungus-farming termite gut microbiome is shaped by acquisition of their co-evolved fungal cultivar. We reveal that the fungus appears to play a relatively minor role in gut microbiome development since the majority of the microbiome is established upon colony founding. This aligns with previous findings that there is extensive complementarity between symbiotic partners and that the microbiome complements fungal and plant degradation. Our work builds on this by demonstrating that these functions expand when the fungus comb is established. Although functional predictions are based on a small sample size, the results remain largely consistent across four distinct pedigrees, underlining that they likely capture patterns across the population and species. Ideally, the impact of fungus acquisition on the microbiome should be tested in other species to confirm consistencies and elucidate variation across the Macrotermitinae.

Functional predictions based on DNA sequencing alone only indicates genetic potential and is unlikely to fully capture biologically relevant processes, as evidenced by the AZCL assays of a small set of CAZymes. Therefore, studies employing transcriptomics are likely to uncover greater variation between timepoints and give a clearer picture of how microbiome functions change over the lifetime of colonies. Such approaches are likely to also reveal changes in other symbiont contributions, as well as elucidate whether specific microbial genes are critical in sustaining colonies at different time points. Moreover, although we successfully recovered a wealth of predicted CAZymes, interpreting their functional roles was limited as many lack substrate target predictions. Future work to improve such predictions would aid in interpretations, as would further characterisation of enzyme activities across the fungus-farming termite phylogeny. Given that most functions are presumably encoded for by the inherited microbiome, and since farming termite diets are broadly comparable, we predict similar principal patterns – including in *Macrotermes bellicosus* and the genus *Microtermes* that transmit *Termitomyces* vertically.

Farming termites are not unique in their fungicultural practices. Functional complementary across the holobiont of insect host, cultivar fungus, and bacterial associations have also been predicted and observed in e.g., fungus-farming ants and ambrosia beetles [27, 64]. Despite distinct differences in traits, convergent evolution has allowed near-identical ultimate functions of these mutualisms – the symbiotic conversion of recalcitrant plant biomass for animal consumption. Fungal cultivars are predominantly vertically transmitted by ant and beetle hosts, while horizontal transfer dominates in termites [22, 65, 66]. As for gut microbiomes, farming termites – as other termites and their ancestral cockroaches – host diverse gut microbiomes that serve a broad suite of functions [15, 29, 67]. Conversely, fungus-farming ants host relatively simple microbiomes that serve an apparently narrower set of critical metabolic functions [36] as is also the case in ambrosia beetles [68]. Symbionts beyond filamentous fungi and bacteria – such as Archaea and yeasts – also contribute and should be integrated in future work of complementary symbiont actions in fungicultural systems. Thus, independent origins and markedly-different evolutionary histories imply potential for future work to decipher distinct building blocks that allow complementary symbiont contributions in extant farming lineages.

### Electronic supplementary material

Below is the link to the electronic supplementary material.

Supplementary Material 1: Table S1 GPS coordinates of field colonies collected in October 2019 in Pretoria, South Africa.

Supplementary Material 2: Table S2. Metadata for metagenomic samples and sequencing information provided by BGI.

Supplementary Material 3: Table S3. AZCL substrates and their enzyme targets. All enzymes are carbohydrate-active enzymes (CAZymes).

Supplementary Material 4: Table S4. Remaining reads after filtering and de-hosting of the metagenomes.

Supplementary Material 5: Table S5. Pairwise statistical comparisons.

Supplementary Material 6: Table S6. TukeyHSD results of ANOVA tests of the effect of timepoint on CAZyme and Nitrogen cycling gene Shannon diversity and Observed richness.

Supplementary Material 7: Table S7. PERMANOVA results of CAZyme and nitrogen cycling gene compositions against independent variables. Distances are Bray-Curtis dissimilarities.

Supplementary Material 8: Table S8. ANOVA of enzyme activities and Tukey HSD pairwise comparisons across timepoints.

Supplementary Material 9: Table S9. Raw data and metadata for AZCL assays across all samples, including averages of three technical replicates.

Supplementary Material 10: Table S10. Raw ddPCR data and metadata. Final unit used in analysis is 16 S *rRNA* copies per gut or 1 cm^3^ fungus comb sample.

Supplementary Material 11: Table S11. 16 S *rRNA* amplicon sequencing sample metadata.

## Data Availability

Amplicon sequences, raw metagenome reads, and assembled metagenomes are available in GenBank via accessions SRR27833599, SRR27833600, SRR27833601, SRR27833602, SRR27833603, SRR27833604, SRR27833605, SRR27833606 ,SRR27833607, SRR27833608, SRR27833609, SRR27833610, SRR27833611, SRR27833612, SRR27833613, SRR27833614, SRR27833615, SRR27833616, SRR27833617, SRR27833618, SRR27833619, SRR27833620, SRR27833621, SRR27833622, and SRR27833623. Raw data for AZCL assays and ddPCR are included as Table S9 and S10 respectively. Metadata used for 16 S rRNA amplicon sequencing can be found in Table S11. All code used for analyses is available on GitHub: https://github.com/Rob-murphys/pre-post_fungus.
